# Pregnant women’s experiences with the management of hypertensive disorders of pregnancy: a qualitative study

**DOI:** 10.1186/s12913-021-07320-4

**Published:** 2021-12-02

**Authors:** Amyna Helou, Kay Stewart, Kath Ryan, Johnson George

**Affiliations:** 1 Centre for Medicine Use and Safety, Faculty of Pharmacy and Pharmaceutical Sciences, Monash University, 381 Royal Parade, Parkville, Melbourne, Victoria Australia; 2 Reading School of Pharmacy, University of Reading, Whiteknights, Reading, RG6 6AP UK

**Keywords:** Pregnancy, Hypertension, Chronic hypertension, Pre-eclampsia, Experiences, Management

## Abstract

**Background:**

Hypertensive disorders are a leading cause of mortality and morbidity during pregnancy. Despite multiple national and international clinical guidelines and a plethora of research in the field of optimising management, there has been limited research describing the perspectives and experiences of pregnant women with the management of hypertensive disorders of pregnancy (HDP). Understanding these perceptions and experiences is imperative to the optimisation of HDP management.

**Methods:**

A qualitative study involving face-to-face, in-depth interviews were undertaken with 27 pregnant women diagnosed with and being treated for HDP to explore their perspectives of and experiences with clinical management. Written consent was obtained individually from each participant, and the interviews ranged from 16 to 54 min. Inductive codes were generated systematically for the entire data set. Line-by-line analysis was then performed and nodes were created within NVivo, a qualitative data management software. Data collection was continued until thematic saturation was reached. Thematic analysis was employed to interpret the data.

**Results:**

Three major descriptive themes were discerned regarding the women’s perspectives on and experiences with the management of HDP: attitudes towards monitoring of HDP, attitudes and perceptions towards development and management of complications, and perceptions of pregnant women with chronic hypertension. Trust in the hospital system, positive attitudes towards close blood pressure monitoring as well as self-monitoring of blood pressure, and a realistic approach to emergency antenatal hospital admissions contributed to a positive attitude towards monitoring of HDP. Women with prior experiences of HDP complications, including pre-eclampsia, were more confident in their clinical management and knew what to expect. Those without prior experience were often in shock when they developed pre-eclampsia. Some women with chronic hypertension displayed limited understanding of the potential risks that they may experience during pregnancy and thus lacked comprehension of the seriousness of the condition.

**Conclusions:**

The clinical management experiences of pregnant women with HDP were varied. Many women did not feel that they were well informed of management decisions and had a desire to be more informed and involved in decision-making. Clear, concise information about various facets of HDP management including blood pressure monitoring, prescription of the appropriate antihypertensive agent, and planning for potential early delivery are required**.**

## Introduction

Hypertensive disorders of pregnancy (HDP) affect around 10% of pregnancies in Australia and around the world [1]. Combined, they are the second largest cause of maternal death, after haemorrhage, in the developed world [1].

In Australia, the public health system provides maternity care from pre-conception to postpartum. The main health professionals who care for the pregnant women are obstetricians, midwives, general practitioners (GP) and obstetric physicians [1]. The GP has an important role in pre-conception counselling, especially with women who have chronic diseases such as hypertension or asthma. It is also the responsibility of the GP to confirm the pregnancy and refer the woman to the relevant maternal hospital service.

Initially, the choice of model of care is given to the woman. The Midwifery Group Practice model [1] allows for one-to-one maternal care, often with the same midwife throughout the term of pregnancy, which is a suitable option for women without complications. Pregnant women with complications such as chronic hypertension or a previous pregnancy complicated by hypertensive disorders of pregnancy (HDP), however, need to be cared for by an obstetrician, who can monitor the progress of the pregnancy, blood pressure (BP), signs of pre-eclampsia, and fetal growth. The obstetric physician is usually involved in prescribing and monitoring antihypertensive medication and BP control. Pregnant women who have had pre-eclampsia previously or who have chronic hypertension are at risk of developing pre-eclampsia. Timely administration of low-dose (81-100 mg) aspirin before 16 weeks gestation has been found to reduce risk of pre-eclampsia [2].

Monitoring of BP occurs at each antenatal visit. If her BP is elevated, the woman may be referred to a day assessment unit for 4-h assessment of BP, which involves taking BP readings every half an hour for 4 h to observe the pattern of the BP and decide whether a diagnosis of HDP and/or prescription of an antihypertensive medication is warranted. In addition, test for urinary protein, full blood examination, renal function tests and fetal monitoring are performed [3]. This 4-h assessment is seen as a favourable alternative to overnight inpatient stays, both in terms of patient satisfaction and public health economics [3].

The timing of delivery in women with HDP is dependent on many maternal and fetal factors, including inability to stabilise BP, deteriorating liver and/or renal function, placental abruption, and severe fetal growth restriction [4]. Fetal morbidity and mortality are linked to the gestational age at delivery [4], so there is always a desire to prolong the pregnancy as close as possible to term (37 weeks) in the absence of an emergency. HYPITAT was a multicentre, open-label randomised controlled trial investigating induction of labour versus expectant monitoring for gestational hypertension or mild pre-eclampsia after 36 weeks’ gestation [5]. The study reported a reduction in the incidence of severe hypertension as result of induction of labour at 36 weeks gestation. No significant clinical differences were found in outcomes such as thromboembolism, eclampsia or placental abruption [5]. This study was followed by HYPITAT-II, which found that delivery should be deferred until 37 weeks as opposed to 36 weeks, unless maternal deterioration supervenes [6].

Despite multiple clinical guidelines [4, 7, 8] and a plethora of research in the field of optimising HDP management, there have been limited published studies describing the experiences of pregnant women with the management of HDP, as distinct from medication treatment.

A survey of women with pre-eclampsia or their partners, friends or relatives found that many had no knowledge of pre-eclampsia prior to diagnosis and once diagnosed, did not appreciate how serious or life threatening it was [9]. Women wanted access to information about pre-eclampsia and their experience contributed substantial anxiety towards future pregnancies. Partners/friends/relatives also had no prior understanding of pre-eclampsia and expressed fear for the woman and/or her baby [9]. A qualitative study of pregnant Moroccan women in the Netherlands or Morocco found that knowledge of symptoms related to hypertensive disorders of pregnancy was limited or absent [10]. The limited knowledge of hypertension-related symptoms and complications was based on their own experiences or on those of some family members or stories from their social network or internet, with little or no information on symptoms from their midwives or obstetricians [10]. The experiences, perceptions and behaviours of pregnant women with regard to the management of HDP during pregnancy remain largely unexplored. Understanding these perceptions and experiences is imperative to the optimisation of HDP management.

## Aim

To explore pregnant women’s perspectives of and experiences with clinical management of HDP.

## Methods

### Study design

A qualitative study using in-depth interviews was conducted, with pregnant women in their second or third trimester, recruited from antenatal clinics in two large tertiary hospitals in Melbourne, Australia.

Participants were sourced via a larger mixed-methods study, which included 100 pregnant women with HDP. Eligible participants were identified by one researcher (AH), who reviewed the medical records of pregnant women attending antenatal clinics, and then approached them individually. Participants were provided with written information for the larger study and on receipt of written informed consent, a questionnaire was given for self-completion. At the end of the questionnaire, participants were asked to indicate their interest in undertaking an interview. Of the 98 women who responded to the questionnaire, 65 expressed interest in being interviewed. Combined convenience and purposive sampling was conducted among these 65 women to seek a breadth of views. All of the women who were invited accepted to participate in an interview. Informed written consent was obtained prior to each interview, which included permission to audio record the conversation and to use quotations when anonymously reporting and publishing the results.

### Study sample

Face-to-face, qualitative in-depth interviews were conducted with 27 pregnant women who were diagnosed with HDP and had a prescription for an antihypertensive medication, in either the second or third trimester of pregnancy, recruited from the antenatal outpatient clinics of two large tertiary maternity hospitals in Melbourne, Australia. Together, these hospitals provide antenatal care to approximately 13,000 women annually. They were identified using hospital records and approached during subsequent clinic visits. Participation was voluntary and involved informed consent.

The study sample size was determined based on theme saturation during analysis and was not predetermined. Recruitment ceased when no new information was forthcoming from the last three interviews, with regard to replication of data relating to attitudes towards HDP monitoring, perceptions of the development and management of complications (including early delivery) and perceptions of the women who had chronic hypertension.

### Data collection

Interviews were conducted face to face by a single researcher (AH) a female Pharmacist (who was a PhD candidate at the time) after receiving training in in-depth interviewing prior to the commencement of the study using an interview guide developed based on the literature [11, 12] and was agreed upon by the authors (Table 1). Open-ended questions, such as “Tell me about …? ”, followed by appropriate prompts, such as “How did that make you feel?” or “Can you explain that in more detail?” were used to guide the interview and encourage the interviewee to speak freely and in-depth about their experiences and thoughts. As the interviewer had met the participants during the larger study, some rapport had been established prior to the interview. The interviewer did not disclose their healthcare background to participants to avoid requests for health advice during the interviews. Interviews on average lasted 35 min (range 16 to 54 min) and were conducted in a private room near the antenatal clinics. No repeat interviews were performed.

**Table 1:** Interview Topic Guide

**Topic one: Understanding of hypertension**	
Explore the women’s health beliefs surrounding their diagnosis of hypertension, e.g. when it was diagnosed and how they felt about it. Exploration into their beliefs regarding causation may also occur.	
**Topic two: Antihypertensive medication use during pregnancy**	
Explore concerns and experiences associated with the safety of using specific antihypertensive medications during pregnancy and thoughts on the importance of continuing them through pregnancy. Investigate whether there was decreased or increased use of any particular medication and why, and factors contributing to compliance. Ask participants to compare the use of blood pressure medications to other medications during pregnancy.	
**Topic three: Medication beliefs**	
Explore the women’s general medication beliefs related to the use of other medications during the current pregnancy, including over-the-counter medications, vitamins and alternative therapies, their perceived safety and benefits.	

Socio-demographic and self-reported health information was collected from participants through the questionnaire. Health information was verified, with consent, through medical records.

All interviews were audio-recorded, transcribed verbatim and de-identified. Interviews continued until data saturation was reached, deemed to be the point after which no new information for analysis was forthcoming [13]. The transcripts were not returned to the participants for comments or correction.

### Data analysis

Data analysis occurred concurrently with the interviews. Initial coding was completed by AH using the qualitative data management software QSR NVivo 10 (QSR International) [14]. Inductive codes were generated systematically for the entire data set. Line-by-line analysis was then performed and nodes were created within NVivo. To ensure reliability, a random selection of 20% of the transcripts were coded independently by another member of the research team (KS). KS and KR read all the transcripts and any differences were discussed among all three to reach consensus. The researchers were all pharmacists; KS and KR had extensive experience in conducting qualitative research. Transcripts were reread by AH and KS to ensure that coding was accurate and all relevant data were included.

Thematic analysis was employed [15]. This was done across all HDP subtypes and severities to obtain a wide range of views. AH read and reread the codes, collapsed them into potential themes, compared the developing themes with the intact transcripts and cross referenced to HDP subtypes. When a pattern was seen within a certain subtype, coding was grouped specifically for that subgroup. Codes were arranged into potential themes. Themes were reviewed, refined and prepared into a final set with KS; sub-themes were identified within this process.

## Results

### Participants

Of the 98 women who responded to the questionnaire, 65 expressed interest in being interviewed. A combination of convenience and purposive sampling was conducted among these women to seek a wide breadth of views. All participants had a diagnosis of HDP and were prescribed antihypertensive medication. Interviews occurred during pregnancy except for one, which happened 1 day postpartum. All participants were aged 18 years or over and were fluent in English. Twenty-seven women were interviewed to reach data saturation. Their demographics, clinical and obstetric characteristics are shown in Table 2. Family members were present for some interviews but none of them participated in the interview or made comments. Field notes were taken by the interviewer during the interviews. No participants dropped out of the study or refused participation.

**Table 2:** Demographics, clinical and obstetric characteristics (*n* = 27)

Characteristics	N
Country of birth	
Australia	18
Other (India, Philippines, Nigeria, Malaysia, Indonesia, United Kingdom)	9
Other health conditions	
None	16
Kidney disease	4
Depression	3
Type 2 diabetes	1
Congenital heart disease	1
Carpal tunnel syndrome	1
Rheumatoid arthritis	1
Gestational stage at interview	
Second trimester	6
Third trimester (32–34 weeks)	5
Third trimester (35–37 weeks)	6
Third trimester (≥37 weeks)	9
1 day postpartum	1
Time of hypertension diagnosis	
Pre-pregnancy	18
Current antihypertensive regimen started during pregnancy	9
< 20 weeks	3
At 20 weeks	0
> 20 weeks	6
Subtype of hypertension^a^	
Gestational hypertension	3
Pre-eclampsia	3
Severe pre-eclampsia	2
Chronic hypertension	10
Secondary hypertension	3
Pre-eclampsia superimposed on chronic hypertension	4
Severe pre-eclampsia superimposed on chronic hypertension	1
Pre-eclampsia superimposed on secondary hypertension	1
Severity of hypertensive disease^a^	
Mild-moderate	20
Severe	7
Antihypertensive medication^b^	
Methyldopa	11
Labetalol	15
Atenolol	2
Nifedipine	1
Oxprenolol	1
Phenoxybenzamine	1

^a^Classification according to the Society of Obstetric Medicine of Australia and New Zealand (SOMANZ) guidelines 2014 [1]

^b^some participants were prescribed more than one antihypertensive medication

Eight participants were primigravidae, the remainder were multigravidae, including six who had previous miscarriages. One participant had an assisted pregnancy (in vitro fertilisation). Twelve were prescribed aspirin for the prevention of pre-eclampsia. Participants ranged in age from 26 to 42 years. The annotations at the end of each quote give a description of the participant’s age, parity, gestational trimester and subtype of HDP at the time of the interview. The participants did not provide feedback on the findings.

### Interview themes

Three major descriptive themes were discerned regarding the women’s experiences with the management of HDP:

- attitudes towards monitoring of hypertensive disorders of pregnancy;
- attitudes and perceptions towards development and management of complications; and
- perceptions of pregnant women with chronic hypertension.

**Theme 1: Attitudes towards monitoring of hypertensive disorders of pregnancy.**

Most women had general trust in the hospital system. Some felt extra confidence knowing that they were being managed at a maternity hospital:

“Hospital is for saving lives of people...as soon as I see the hospital I know that I am in safe hands.” (#14, 40 years, 2nd pregnancy, second trimester, severe pre-eclampsia).

“I felt very comfortable here...it seems like they are well prepared for these things...I was in the section of the hospital where all women were in the same [hypertension] situation.” (#9, 30 years, 1st pregnancy, second trimester, chronic hypertension).

One woman expressed some distrust in general hospitals, which she perceived as not managing her BP well:

“My own GP at that time increased it [methyldopa] to six a day … the hospital … increased to 10 a day … but I couldn’t lift my head up … so I ended up coming to the women’s hospital to Emergency, because I felt like no one’s helping me.” (# 71, 37 years, 3rd pregnancy, third trimester, severe pre-eclampsia superimposed on chronic hypertension).

Self-monitoring of BP was often recommended to women treated with antihypertensives. For some, it gave reassurance, but for others it was a source of confusion, with different messages coming from various members of the treating team:

“I take up to eight [methyldopa tablets] a day...I take two and then I’ll see what my readings are...at home, myself...I also do it if I have any other symptoms.....” (# 71, 37 years old, 3rd pregnancy, third trimester, severe pre-eclampsia superimposed on chronic hypertension).

“One of the physicians I saw told me to do it three times a day … three times in a row and then she said take the average of the second two readings each time...Then I told the obstetrician my readings and she said the machine that I had at home is under-measuring...but...the physician, he was quite interested...he wanted to see my readings because he likes to compare his machine to home machines.” (#53, 40 years, 6th pregnancy, third trimester, secondary chronic hypertension).

Some women had milder HDP in previous pregnancies, which gave them a sense that the monitoring and management was overstated. Others with more severe cases and their prior experience brought back memories initiating action to make plans:

“With [child 1] it was really bad during pregnancy. With [child 2] it was bad just in the last couple of weeks and straight afterwards...[child 3] was bad, but not really bad enough. They just called it hypertension, pregnancy induced hypertension and they just left it at that. They didn’t make a big song and dance about it...They made a big song and dance last time [child 4] and then this time [they said] ‘You’re going for your monitoring’...come back a week later...’You’re going for your monitoring’, couple of hours later – ‘You’re being admitted’...Then a couple of hours later...’You’re starting on medicine’.” (#6, 28 years, 4th pregnancy, 1 day postpartum, gestational hypertension).

“Last Wednesday when they [found] blood pressure’s up … it just brought back memories from last time because same thing. I just went in for an appointment and I never came home...Ten days later I came home with a baby. So I think those aspects freak me out a bit because it’s like ‘Oh, it’s happening again’ … every appointment...even this appointment, we’ve got contingency plans, just in case.” (#74, 36 years, 2nd pregnancy, third trimester, chronic hypertension).

Some women wondered about why they were not told their BP readings unless they asked:

“I find it funny that when they take your blood pressure they don’t tell it to you. I always have to ask, always, no matter who it is, midwife, physician, obstetrician. They take it and they walk away. It’s my body but they don’t tell me.” (#53, 40 years, 6th pregnancy, third trimester, secondary chronic hypertension).

Close monitoring was perceived as frustrating, but also as part of the life adjustments that come with having a baby. Some women considered spending four hours in the day assessment centre for monitoring their BP better than being an inpatient and staying overnight, whilst others saw it as an annoyance:

“I am happy to come back every day as long as I don’t have to spend overnight here. I am happy to be here for 12 hours a day, but I just can’t be away from my children at night time.” (#29, 26 years, 3rd pregnancy, second trimester, secondary chronic hypertension).

“They just monitor me at that perinatal care...you just sit here four hours a day...it’s shocking...worse than taking the tablet.” (#90, 35 years, 7th pregnancy, third trimester, chronic hypertension).

“The only thing that was slightly frustrating was [that] four hours is a long time to sit around, but again, you’re having a baby so you’ve got to make a few adjustments to your life.” (#99, 34 years, 1st pregnancy, third trimester, pre-eclampsia).

Some of the women required a short inpatient stay to stabilise their BP and avert an emergency premature delivery. For many, it was an emotional experience filled with apprehension and uncertainty about the future:

“It was very emotional, very scary, and at the same time still trying to stay strong. So that when my husband and my kids came in, I was like ‘I’ve just got a little bit high blood pressure, everything’s alright’...I didn’t know that a possible side effect of having the blood pressure is that they may have to deliver the baby [early].” (#32, 42 years, 3rd pregnancy, third trimester, severe early onset pre-eclampsia).

“B.P. at first was around 160...she came back 15 minutes – 170, another 15 minutes 180, within 10 minutes 190...I got nervous … After 160 they gave me ...labetalol...but [the BP] did not go down...There were other tablets they gave to me but [the BP still] didn’t go down...All the doctors came up...surrounded with those with scrub suits, I panic...blood pressure...went to 210..they were panicked...one just looked at me and said “AAAH”. I cried...of course you feel anxious, you feel sad...worried... what’s going on with me? I cried and cried. It was just like a movie, they push my bed out from the room and sent me quickly down to the birthing suite [in case delivery was imminent].” (#14, 40 years, 2nd pregnancy, second trimester, severe pre-eclampsia).

**Theme 2: Attitudes and perceptions towards development and management of complications.**

For many women, the diagnosis of pre-eclampsia came as a shock. Those with prior experience knew what to expect and were hesitant to cease antihypertensive treatment even if their BP was low. One woman without prior experience self-educated about pre-eclampsia, became concerned about the symptoms and developed anxiety about developing it:

“It was a shock and it was a bit scary...I thought ‘I’ve heard of pre-eclampsia but I don’t really know what it is’...but all the staff, they explained everything quite well... [I could see] how they were being very concerned about it, so that was making me realise this isn’t just a small thing, this is obviously a serious situation.” (#32, 42 years, 3rd pregnancy, third trimester, severe early onset pre-eclampsia).

“I didn’t want to stop the medication altogether, only because I just didn’t want to go through the path of having the high blood pressure affect the baby [intra-uterine growth restriction].” (#8, 36 years, 2nd pregnancy, third trimester, chronic hypertension).

“I was reading that if you do develop pre-eclampsia... it is a risk for the baby and the mother as well. Upon reading all that information...I became a bit paranoid, swollen foot, swollen hands, they’re part of the symptoms, headaches, generally not feeling well...I became quite paranoid looking at my symptoms and [thinking] have I got this, have I not got this? But the doctors actually did say that I have got borderline pre-eclampsia, so they were waiting to see if I was going to develop it. However, they haven’t been able to reassure me that I’m not going to develop it and...that was quite scary for me.” (# 24, 35 years, 1st pregnancy, third trimester, chronic hypertension diagnosed during pregnancy).

Some women understood that low-dose aspirin was being used for prevention of pre-eclampsia, whereas others did not always perceive it as being effective for this purpose. Many women thought that aspirin helped with controlling the BP rather than for prevention of pre-eclampsia:

“I started on aspirin throughout the pregnancy … just to...prevent mild pre-eclampsia happening again.” (#64, 30 years, 3rd pregnancy, third trimester, chronic hypertension).

“Obstetrician put me on one aspirin a day which is supposed to help control blood pressure. So perhaps that’s also why my blood pressure is being well controlled.” (#21, 35 years, 1st pregnancy, second trimester, chronic hypertension).

Most women had a general understanding that the only way to stop the direct effects of pre-eclampsia was to deliver the baby for the safety of both mother and child. The level of comfort with such a decision varied depending on the gestational stage of diagnosis of pre-eclampsia:

“I was really disappointed and very worried about the effect it [pre-eclampsia] would have on the baby [at 21 weeks] and whether or not I would be able to carry the baby to a safe week. I just thought...if something had happened and I was forced, like accidentally went into labour too early or something like that, the baby’s chances of survival would be very low and I was really upset.” (#21, 35 years, 1st pregnancy, second trimester, chronic hypertension).

“All I know is that you just need to get the baby out...I mean plenty of women and plenty of babies survive it...but you need to detect it pretty quickly before it turns into the full...is it eclampsia?” (#41, 34 years, 1st pregnancy, third trimester, chronic hypertension).

Although many women understood that they would not continue to full-term, their perceptions and fears about the potential for a premature delivery were related to their week of gestation, concern about the welfare of the baby, and fear of separation after the birth:

“I know from my reading that 24 weeks, it’s still not ideal obviously, but if you had the baby at 24 weeks that the chance of survival was higher. I think it was 43% chance of survival from this...prior to that it was like 16% chance of survival...My sister-in–law, who is a midwife, had said...they consider 26 weeks more viable. So after that it was like, right (a) to get to 24, (b) get to 26.”. (#21, 35 years, 1st pregnancy, second trimester, chronic hypertension).

“I am just worried about my baby [having] to be delivered earlier because you see the consequences...you see things happen in the future...they are still very weak...no sucking reflex yet, the lungs are not fully developed, so many things not developed...she may live but maybe there are some disabilities...I am just hoping that I will reach even up to 30 weeks or 32 weeks. That would make me feel better.” (#14, 40 years, 2nd pregnancy, second trimester, severe pre-eclampsia).

“I was 28 weeks [when I developed severe pre-eclampsia]...they gave me steroid injections to increase the lung capacity of the baby...One of the doctors came from the NICU with a leaflet about possibly having a premature baby...that was very upsetting...and to think of having the baby...then me going home with the baby staying here is just a very scary thought.”(#32, 42 years, 3rd pregnancy, third trimester, severe early onset pre-eclampsia).

Intervention with the delivery process was a likely reality for many women who had a prospect of early delivery. Some women were apprehensive about the prospect of induction of labour or caesarean section but understood that it was for their benefit and that of their child. Others were hesitant to allow for intervention unless the risks were made clear:

“So a little bit scary, but in a way I want it to, because I’m starting to feel the uncomfortable risk that’s associated with pregnancy in this condition. Knowing that she’s at full term now at 37 weeks and she’s fine and healthy, I don’t want to develop pre-eclampsia if I can help it.” (# 24, 35 years, 1st pregnancy, third trimester, chronic hypertension diagnosed during pregnancy).

“I’m trying to push it off because I don’t want to do it. I like to have the baby when the baby’s ready, not when they tell me to. But if they tell me to because it’s really dangerous for me then I’ll listen to them obviously ….” (#53, 40 years, 6th pregnancy, third trimester, secondary chronic hypertension).

Concerns about lack of information sharing by health professionals led some women to feel that they were left out of the planning for potential intervention in the delivery, whilst others voiced concern about having low-dose aspirin in the context of a possible emergency caesarean section:

“I even asked her last time actually because she said...I’m happy with the baby’s growth, but the blood pressure’s going up so … she said ‘I’m formulating a plan in my mind’ but she doesn’t like to disclose it. I don’t know why. It’s about me; I don’t know why she just doesn’t tell me.” (#53, 40 years, 6th pregnancy, third trimester, secondary chronic hypertension).

“I also thought...what if I have an emergency caesarean tomorrow and I haven’t gotten off the aspirin? Is it going to cause me issues?” (#29, 26 years, 3rd pregnancy, second trimester, secondary chronic hypertension).

**Theme 3: Perceptions of pregnant women with chronic hypertension.**

Many women with chronic hypertension were already on antihypertensive medication not deemed safe during pregnancy when they found out they were pregnant. For some, it was changed to a safer alternative as soon as possible, whilst for others, the decision to change the medication was delayed and the patient’s assessment of potential risks was downplayed:

“I was on medication [telmisartan]...then when I had the kidney scan and I found out [that I was pregnant], my G.P. said ‘You’ve got to stop taking that medication because it’s not safe...so then she gave me another one to take.” (#2, 30 years, 1st pregnancy, third trimester, secondary chronic hypertension).

“The first time I found out I was pregnant I went to a GP...I told the GP that I’m taking atenolol, and then she told me that...atenolol is not recommended for pregnancy... so I asked....What medication do you think that I should take?’...she said she doesn’t dare to prescribe me any medicine because she knows she is going to refer me to a hospital.” (#59, 34 years, 1st pregnancy, second trimester, chronic hypertension).

Other women had their antihypertensive changed during the pre-pregnancy planning stage:

“[To be safe during pregnancy] I would just have to change my medications. The medication I was on I couldn’t be on while being pregnant. So when we decided to try for our first child, I went on the Aldomet and oxprenolol and that’s what I pretty much stayed on because we always wanted a second child.” (#1, 39 years, 2nd pregnancy, third trimester, secondary chronic hypertension).

Some women with chronic hypertension had concerns about lack of information sharing by health professionals and felt that they were not well informed of the potential risks that their hypertension may have on the pregnancy. Some mentioned that they may have ‘taken it more seriously’ if they had known about the risk of premature delivery associated with uncontrolled hypertension, whilst others had some limited awareness of pre-eclampsia:

“When they told me I had protein in my urine, I was a bit scared because I don’t know if it’s related to my BP.” (#4, 33 years, 1st pregnancy, third trimester, chronic hypertension).

One woman was very anxious about her diagnosis of severe, early-onset pre-eclampsia so she did some ‘self-research’. Unfortunately, she misinterpreted the information and caused herself extra unwarranted fear:

“I read on [US website found on Google] and found that 80% die after/during birth that have pre-eclampsia. That was really scary.” (#71, 37 years, 2nd pregnancy, third trimester, severe pre-eclampsia superimposed on chronic hypertension).

The information on the US Preeclampsia Foundation website actually states that “Nearly 80% of women who die from pre-eclampsia die post-partum” [16].

For some women with chronic hypertension, lack of knowledge of the seriousness of the condition resulted in lack of comprehension of the importance of BP monitoring and treatment:

“I think it was about 140 over 110 or something like that … which is pretty normal for me but they think it’s high … I feel alright. It’s all good.” (#90, 35 years, 7th pregnancy, third trimester, chronic hypertension).

“I really tried for weeks not to go on [the antihypertensive], but then when she said that maybe you could have a stroke, I got a bit scared, a lot scared … I got really worried because then they said … you could have problems, the baby could die. And I got really upset when she said the baby could not get enough oxygen. I just felt, oh just have whatever it is.” (#22, 37 years, 3rd pregnancy, third trimester, chronic hypertension).

Most women who had chronic hypertension were under a model of care involving both an obstetrician and a physician. One was triaged to midwife-only care, despite having a diagnosis of chronic hypertension and being prescribed an antihypertensive medication. This then caused a delay in the change of the antihypertensive to a safer alternative:

“Actually, I asked the midwife whether it [atenolol] is safe or not [at 18 weeks gestation]...and then she said that...it should be okay, but to be safe discuss with the physician. And so, because she said it should be okay, I presumed that ‘Oh that is okay’**...**but then the physician said ‘No, it’s better not to...so from now onwards you have to take this medicine [oxprenolol]’.” (#59, 34 years, 1st pregnancy, second trimester, chronic hypertension).

Many women had their first antenatal appointment at the hospital between 16 and 20 weeks gestation. Some women, especially those with chronic hypertension, had concerns about the timing of this appointment:

“It takes a long time now for women to get their first appointment through the hospital. It wasn’t like that, I think, about 10 years ago, must’ve changed by now … Now you have to wait ‘til you’re about 18, 20 weeks before you get your first actual appointment...and if you’ve got other health issues, things can go wrong, which it did with me.” (#71, 37 years, 2nd pregnancy, third trimester, severe pre-eclampsia superimposed on chronic hypertension).

Some women did not know that they had high BP before pregnancy. This may have been because they did not get regular check-ups with the GP or that their BP was not routinely checked at regular GP visits:

“I think if I had never gotten pregnant, I definitely would not have had [high BP], would not have to be on medication … because I wouldn’t be under the strain that I am. And also I wouldn’t be in with the doctor. I don’t think I would’ve gone to the doctor and said put me on medication … because I didn’t, want anything to change. But my lifestyle is changing now so I don’t have a choice.” (#18, 35 years, 2nd pregnancy, second trimester, chronic hypertension).

“It [BP] was quite normal before the pregnancy, so obviously it’s pregnancy-related according to the doctors [despite having been diagnosed at 7 weeks].” (#24, 35 years, 1st pregnancy, third trimester, chronic hypertension diagnosed during pregnancy).

Many women had developed chronic hypertension after a previous pregnancy that involved either gestational hypertension or pre-eclampsia. Some of them had routine follow-up for their hypertension postpartum and understood that it was now chronic hypertension, whilst others did not:

“Once I’d had the baby they changed my medication to the perindopril...I was then checking my BP at home...the readings were fine...when they did get too high, I’d go back to my local GP who would then once again adjust the dosage accordingly...I have been told by my local GP that generally once you’re on a blood pressure medication, you’re on it for life, whether it’s a minimal dosage or, depending on what the readings are, what they need to give...I’m happy to stay on that.” (#8, 36 years, 2nd pregnancy, third trimester, chronic hypertension).

“I got increased blood pressure at the end [of the previous pregnancy] and they put me in perinatal care, but then afterwards it was okay....I honestly just didn’t go to the doctor, and I haven’t gone to the doctor since I fell pregnant with this one.” (#90, 35 years, 7th pregnancy, third trimester, chronic hypertension).

One woman described having been prescribed an antihypertensive during her previous pregnancy and never told to stop it, so she continued with no formal review of her hypertension until the current pregnancy:

“They never told me to stop taking the tablet [labetalol] after I had him [first child] so I just kept continuing with it**...**I saw the physician [during this current pregnancy] and he just said just keep taking it...he actually questioned ‘Did they ask you to stop it?...I said no one spoke to me about anything...I was here for a week after I had him [first child]...no one ever discussed it.” (#58, 38 years, 2nd pregnancy, third trimester, chronic hypertension).

## Discussion

Trust in the hospital system, positive attitudes towards close BP monitoring as well as self-monitoring of BP (SMBP) and a realistic approach to emergency antenatal hospital admissions contributed to a positive attitude towards monitoring of HDP. Most of the women in our study had a general trust in the healthcare system. Distrust surfaced when health services outside the women’s hospital were not seen as able to control hypertension early in the pregnancy, triggering patient-initiated referral to the women’s hospital. Trust of healthcare systems in western countries is generally declining [17]. It is, however, important to note that pregnant women with HDP are considered to be in a high-risk pregnancy and are thus more vulnerable than the general population. Therein lies dependence on the hospital system, especially in urgent situations such as needing to lower BP or planning for an early delivery, similar to the dependence reported in patients with coronary heart disease [18].

Anecdotally, it is common for healthcare professionals to mention that the BP reading is ‘good’ or ‘too high’ without telling the patient the systolic/diastolic numbers. An important factor relating to patient evaluation of care is their involvement in decision-making [19]. Most of the women in our study were not involved in decision-making, leaving some to wonder why this was so. This suggests that pregnant women with HDP would like to be better informed of their situation and be part of the decision-making process when deemed appropriate. This is consistent with the women’s views from the pilot of the CHIPS study who enjoyed being heavily involved in their BP management [20]. One way to have women more involved in their BP management is to encourage SMBP. SMBP in the general population has been shown to reduce BP [21] and improve adherence to antihypertensive medication [22]. In our study, SMBP was often recommended to women who were prescribed antihypertensive medications. This was taken up well by most, similar to the CHIPS pilot study [20]. SMBP during pregnancy has also been shown to be reassuring and not anxiety provoking [23] which was seen in our study. A recent survey of 5555 pregnant women from antenatal clinics in 16 hospitals in England, found that nearly half of the 389 hypertensive women reported SMBP, and that the majority of them (79%) shared their BP readings with their treating doctor [24]. Such partnership has been shown to improve patient adherence in the general population [25]. There is however an assumption that because these women are in a high-risk pregnancy, the healthcare professionals (HCP) tend to take over and do not acknowledge that the women are quite competent and that with the correct information can be involved in SMBP in collaboration with the HCPs. It is thus important to have a good doctor-patient relationship to reduce confusion, instil confidence in SMBP and complete the circle of care.

Those with prior experience with HDP and monitoring had varied views, often depending on the severity of disease in the previous pregnancy (ies). Good communication about how HDP can vary from one pregnancy to another, being either worse or better, may assist in reducing the cynicism of some and reassure others. Similarly, those who had prior experience with pre-eclampsia were a lot more confident in their management and knew what to expect. Those who did not have prior experience were often in shock and were at times anxious about the diagnosis, a finding similar to another study relating to the understanding of pre-eclampsia [26]. Moreover, the use of low-dose aspirin to prevent pre-eclampsia was only partially understood by women in our study. This indication should be communicated clearly to women who are at high risk of developing pre-eclampsia. The plan for the cessation of aspirin before delivery should also be communicated clearly to reduce any anxiety that may be present, especially in terms of a potential emergency delivery.

At times, antenatal inpatient admission was required to stabilise BP and closely monitor both the mother and baby. This was a particularly apprehensive time, especially for women who had not experienced HDP during a previous pregnancy. It is important to have good, clear communication with women about the need for close monitoring, affirmation concerning their status as worthy of hospital care, provision of consistent information, inclusion in decision-making and good social support [27]. A possible alternative to inpatient admission can be pregnancy day assessment monitoring. Despite limited research into this model of care, pregnant women have been found to prefer a four-hour stay rather be admitted to hospital for one or more nights, if the situation is deemed safe to do so [3]. This is consistent with our findings. Once again, clear consistent information regarding the need for this type of monitoring should be given to women who require it. Recent advancements in the integration of telemedicine into antenatal care [28] have encouraged early research into the feasibility of incorporating this for women with HDP to reduce the burden of multiple antenatal hospital visits [29]. The unpredictable course of worsening BP and the development of pre-eclampsia pose specific challenges to this monitoring and would require a holistic approach. A recent single centre study in the UK [29] developed and trialled an innovative SMBP intervention including a downloadable mobile app in for women with HDP to monitor for signs of pre-eclampsia or worsening hypertension. Although this study showed positive acceptance and compliance from the women, further research is required to meet the standard of care required for them [29].

In general, most women desire to labour spontaneously and have a natural birth [30]. When the reality that the only way to stop the direct effects of pre-eclampsia is to deliver the baby at any given gestational week is revealed to some women, it is received with disappointment. Good communication by the treating doctor about the intention to preserve the pregnancy for as close as possible to term is required. Likewise, sound communication about the need for a premature delivery should be communicated clearly. Moreover, it has been shown that pregnant women who require induction of labour or caesarean section often feel left out of the decision-making process [30]. An Australian study of women’s experiences of decision-making and attitudes in relation to induction of labour, reported a clear need for women to be provided with more information and agency when making decisions about their timing of birth, particularly when there are multiple reasonable treatment options [30]. Furthermore, emergency caesarean sections have been found to negatively contribute to several psychosocial outcomes for women, in particular post-traumatic stress [31]. There is, thus, a need for careful consideration and counselling for women after an emergency caesarean delivery. This can involve the members of the antenatal treating team but also counsellors or psychologists. Moreover, counselling of pregnant women who are at risk of emergency caesarean, either because of their HDP or otherwise, about this possibility may help to pre-empt potential trauma.

Research into the management experiences of pregnant women with chronic hypertension, as distinct from medication treatment, is scant. Our study has highlighted the need for extra attention to be given to improve management pre-conception, during the pregnancy and postpartum. A qualitative study exploring knowledge and attitudes related to pregnancy and preconception health in women with chronic medical conditions, including chronic hypertension, found that the women had limited knowledge of the specific potential complications of pregnancy [32]. Some women in our study also displayed limited understanding of the potential risks that they may endure during pregnancy and thus had a lack of comprehension of the seriousness of the condition. Counselling women pre-conception regarding potential risks during pregnancy allows them to be more aware of what to expect [33]. Moreover, an open conversation about the information that the pregnant woman may have either from prior experience or ‘self-research’ would help to improve understanding as well as avoiding confusion and unnecessary anxiety. The provision of written material so that the women can refer to it when necessary would also be appropriate. Another facet of management of chronic hypertension pre-conception is the initial diagnosis of hypertension. Some women in our study were not aware of their BP readings before pregnancy and were diagnosed with hypertension quite early in pregnancy and thus classed as having chronic hypertension. Regular checking of BP in women of reproductive age at routine GP visits may help to identify chronic hypertension earlier. This can help in the planning of a pregnancy, or ensure that the BP is under control in the case of an unexpected pregnancy. Similarly, for those who have chronic hypertension and are prescribed antihypertensives, switching the medication to one that is safer during pregnancy, either pre-conception or as early as possible, can help to reduce fetal exposure and reduce the mother’s anxiety. Both GPs and community pharmacists have a role in counselling women of reproductive age who are prescribed an antihypertensive. A simple question as to whether the women is planning a pregnancy can help initiate the necessary conversation and trigger the switch to a safer alternative in a timely manner. Various resources are available to help make the decision to change the antihypertensive, including drug information lines at maternity hospitals. Moreover, a large number of women who had entered our study with chronic hypertension had developed the condition after a previous pregnancy that was affected by gestational hypertension or pre-eclampsia. Furthermore, our study found that many women developed chronic hypertension soon after a pregnancy complicated by HDP. This is supported by a recent systematic review and meta-analysis which reported that the risk of developing hypertension after HDP is highest in the early postpartum period [34]. The authors also suggested that diagnosis and targeted interventions to improve maternal cardiovascular health may need to be commenced in the immediate postpartum period [33]. We agree with this and call for a more integrated follow up with women in the postpartum period and beyond. This may involve the GP and the community pharmacist for easy accessibility for the women.

Although most women with chronic hypertension in our study were under a model of care involving an obstetrician and a physician, one was under midwifery care despite having chronic hypertension and being prescribed an antihypertensive. Although it is recognised by Australian guidelines for the management of HDP that midwives play a role in a multidisciplinary team in relation to management of HDP [4, 35], they are not qualified to independently prescribe medication and manage cases of pregnant women with chronic hypertension requiring treatment. Similarly, a recent scoping review found that practising midwives worldwide lack knowledge on several aspects of pre-eclampsia diagnosis and care and have called for an increase in in-service training to increase midwives’ knowledge in this area [36]. It is therefore important that all pregnant women who have chronic hypertension be under the doctor model of care to monitor both mother and baby throughout the pregnancy.

### Recommendations for practice

Good communication between the HCP and the patient is important to optimise management. Clear, direct and concise information about various facets of the management of HDP should be provided for all women who experience HDP.

Women who experience any form of HDP during pregnancy should be invited to be part of the decision-making pertaining to the monitoring of BP and progression to pre-eclampsia as well as the timing and mode of delivery when appropriate.

There should be a priority for women with chronic hypertension to be seen at the hospital under an obstetrician led model of care before 18 weeks, not only for the regular monitoring of BP and fetal progress but also for the timely prescription of low-dose aspirin before 16 weeks gestation for the prevention of pre-eclampsia. Furthermore, other health professionals, including psychologists and pharmacists, can be involved in the prenatal care of these women to address potential fear and anxiety as well as the optimal use of medication.

Women who have chronic hypertension and are of reproductive age should be informed of the potential risks of pregnancy and be switched to a pregnancy safe antihypertensive in the preconception stage.

Women who experience gestational hypertension or pre-eclampsia during pregnancy should have their BP monitored postpartum by the GP or the community pharmacist to identify any risk of developing severe cardiovascular events.

### Recommendations for future research

Currently, much of the monitoring of HDP requires a hospital visit. Further research into the feasibility of telehealth for the monitoring of HDP, especially in mild cases will help to include patients in the decision-making. Moreover, future research into the role of the GP and the community pharmacist in the pre-pregnancy planning stage for those with chronic hypertension and postpartum for those with gestational hypertension or pre-eclampsia is warranted.

### Strengths and limitations

This qualitative study is the first to use in-depth interviews to explore pregnant women’s experiences, perceptions and behaviours with regard to the management of HDP during pregnancy. Our study included women with all forms of HDP except HELLP and eclampsia, as the interviews were done when the women were in a comfortable, non-emergency situation. Participants varied in gestation stage, subtype of HDP, severity of HDP, ethnicity and socioeconomic status allowing for a wide range of views. The interviews were conducted during pregnancy thus reducing recall bias. This is in contrast to other qualitative studies which explored aspects of HDP in retrospect [27, 35, 37]. Recruitment was from two major public maternity referral hospitals in Melbourne with a widespread combined catchment including metropolitan, regional and rural areas. Participants did not include those in the first trimester of pregnancy, as most were scheduled to attend the antenatal clinics after 12 weeks gestation. Views of women with chronic hypertension during the first trimester of pregnancy may vary from those in the second and third trimesters and they were not captured**.** Women with poor English skills were excluded from the study, therefore, caution should be taken in the extrapolation of our findings to women from non-English speaking backgrounds.

## Conclusions

The clinical management experiences of pregnant women with HDP were varied. Many women did not feel that they were well informed of treatment and management decisions and had a desire to be more informed and more involved in decision-making. Clear, concise information about various facets of HDP management including BP monitoring, administration of low dose aspirin in women with a high risk of developing pre-eclampsia, prescription of the appropriate antihypertensive, and planning for potential early delivery are required. In addition, cardiovascular pre-pregnancy planning and postpartum follow-up should be routinely offered to women.

## Data Availability

The datasets generated and/or analysed during the current study are not publicly available due to privacy surrounding participant information as stipulated in the written consent form, but are available from the corresponding author on reasonable request.
